# The role of oral microbiome in periodontitis under diabetes mellitus

**DOI:** 10.1080/20002297.2022.2078031

**Published:** 2022-06-03

**Authors:** Han Qin, Guangyue Li, Xiaohui Xu, Chuangwei Zhang, Wenjie Zhong, Shihan Xu, Yuanyuan Yin, Jinlin Song

**Affiliations:** a College of Stomatology, Chongqing Medical University, Chongqing, Unknown, China; b Chongqing Key Laboratory of Oral Diseases and Biomedical Sciences, Chongqing, Unknown, China; c Chongqing Municipal Key Laboratory of Oral Biomedical Engineering of Higher Education, Chongqing, Unknown, China

**Keywords:** Diabetes mellitus, periodontitis, periodontal disease, subgingival microbiome, oral microbiome, next-generation sequencing

## Abstract

Periodontitis is among most common human inflammatory diseases and characterized by destruction of tooth-supporting tissues that will eventually lead to tooth loss. Diabetes mellitus (DM) is a group of metabolic disorders characterized by chronic hyperglycemia which results from defects in insulin secretion and/or insulin resistance. Numerous studies have provided evidence for the inter-relationship between DM and periodontitis that has been considered as the sixth most frequent complication of DM. However, the mechanisms are not fully understood yet. The impact of DM on periodontitis through hyperglycemia and inflammatory pathways is well described, but the effects of DM on oral microbiota remain controversial according to previous studies. Recent studies using next-generation sequencing technology indicate that DM can alter the biodiversity and composition of oral microbiome especially subgingival microbiome. This may be another mechanism by which DM risks or aggravates periodontitis. Thus, to understand the role of oral microbiome in periodontitis of diabetics and the mechanism of shifts of oral microbiome under DM would be valuable for making specific therapeutic regimens for treating periodontitis patients with DM or preventing diabetic patients from periodontitis. This article reviews the role of oral microbiome in periodontal health (symbiosis) and disease (dysbiosis), highlights the oral microbial shifts under DM and summarizes the mechanism of the shifts.

## Introduction

Periodontal diseases comprise a wide range of inflammatory conditions of periodontal supporting tissues including gingiva, alveolar bone and periodontal ligament [1]. Gingivitis is the localized inflammation of the gingiva, while periodontitis is characterized by the loss of gingiva, alveolar bone and periodontal ligament. The deep periodontal ‘pocket’ is a hallmark of the disease and can eventually lead to tooth loss [2]. Periodontal diseases are currently considered to share a similar aetiopathogenesis, which is initiated and sustained by the oral microbial biofilm [2]. Other factors such as gene susceptibility and environmental conditions also influence the morbidity of the diseases [3]. Moreover, recent evidence has indicated that periodontitis is epidemiologically associated with several systemic disorders such as atherosclerosis, adverse pregnancy outcomes, rheumatoid arthritis, aspiration pneumonia, certain cancers and diabetes mellitus [4].

Diabetes mellitus (DM) is a group of metabolic disorders characterized by chronic hyperglycemia which results from defects in insulin secretion and/or insulin resistance over a prolonged period of time. Generally, there are two main types of DM: type 1 diabetes mellitus (T1DM) and type 2 diabetes mellitus (T2DM) [5]. T1DM is due to autoimmune β-cell destruction, usually leading to absolute insulin deficiency, including latent autoimmune diabetes of adulthood [5]. T2DM is due to a progressive loss of adequate β-cell insulin secretion and insulin resistance [5,6]. According to the World Health Organization, DM currently affects approximately 422 million people globally and 1.6 million deaths are directly attributed to DM each year. In 2045, the number of diabetic patients is expected to increase to 629 million [7]. Of all the diagnosed diabetes cases, T2DM accounts for 90%-95% [5] and affects more than 380 million people worldwide, representing 8.8% of individuals aged 20–79 years [8]. Moreover, T2DM may cause long-term complications including retinopathy, nephropathy, peripheral neuropathy and atherosclerotic cardiovascular, peripheral arterial and cerebrovascular diseases [9]. Also, periodontal diseases are highly likely to occur and aggravate in individuals with DM especially in poorly controlled diabetics [10]. Likewise, since periodontal diseases may contribute to the body’s overall inflammatory burden, individuals with periodontitis are more potentially to develop DM [2]. Thus, a ‘two-way’ relationship between the two diseases is established [10].

The impact of DM on periodontal diseases through hyperglycemia and inflammatory pathways is well described [10–12], while the effects of DM on oral microbiome remains controversial. Previous studies failed to reach a consensus on that DM affects the oral microbiome [13], possibly because of the limited numbers of bacteria being surveyed [14]. In recent years, the development of next-generation sequencing (NGS) technologies allowed us to study the oral microbiome more comprehensively and their increasing affordability also facilitated such studies to become the mainstream. Thus, the use of NGS has broadened and deepened our understanding about whether DM may exert a selective pressure on oral microbiome by comprehensively comparing the oral microbiome from nondiabetic and diabetic individuals with and without periodontitis. In the next sections, we will review the oral microbiome in health and periodontitis, highlight the alteration of oral microbiome especially subgingival microbiome under DM and summarize the mechanism by which DM changes oral microbiome. The search strategy and inclusion criteria are as follows: search keywords are ‘oral microbiome’ or ‘subgingival microbiome’, ‘diabetes’ or ‘diabetes mellitus’, ‘periodontitis’ or ‘periodontal diseases’ and ‘next-generation sequencing’; search databases include MEDLINE and WEB OF SCIENCE; inclusion criteria are human studies or animal studies on oral microbiome in diabetics by using next-generation sequencing technology and comparing with non-diabetics.

## Oral microbiome and periodontitis

The concern on oral biofilms has been over three hundred years since Antony van Leeuwenhoek peered and observed the existence of microbes from his own dental plaque with a microscope that he constructed himself in the 1700s [15]. With the development of technology, quantities of microorganisms have been isolated from the oral cavity and extensively studied by using cultivation and non-traditional molecular-based approaches [16]. More recently, the next-generation sequencing technology enables microbiologists to study the microorganisms at a specific niche as a whole in unprecedented detail [17]. Consequently, the term ‘microbiome’ was proposed ‘to signify the ecological community of commensal, symbiotic, and pathogenic microorganisms that literally share our body space and have been all but ignored as determinants of health and disease’ by the Nobel laureate microbiologist, Joshua Lederberg [18]. The Human Oral Microbiome Database (HOMD) contains comprehensive information about approximately 775 prokaryote species including both cultivable and non-cultivable isolates that inhabit in the oral cavity, links sequence data with phenotypic, phylogenetic, clinical and bibliographic information and provides tools for use in understanding the role of the microbiome in health and disease [19].

Due to the local environmental features of the oral cavity, members of the oral microbiome co-aggregate and interact with each other by synergism, signaling or antagonism to best adapt to the surrounding environment [20]. Meanwhile, the oral microbial communities interact with host, affecting the oral health of the host. Normally, the oral microbial communities are stable and symbiotic in healthy individuals with healthy food habits and good hygiene, maintaining homeostasis with the host’s local immune system [21–23]. Once the balance was interrupted by external factors such as food habits, tobacco and alcohol consumption, stress, hormonal imbalance, puberty, poor oral hygiene and diabetes, the microbial communities dramatically shift from a symbiotic state to a dysbiotic state, which induces diseases such as periodontitis [16,24,25]. The dysbiotic microbiome can be characterized by three different scenarios that are not mutually exclusive and may occur simultaneously, viz, the overall loss of microbial diversity, relative reduction of the beneficial species and increase of the pathogenic species [26,27].

The concept of loss of biodiversity indicates a decline of richness, numbers and distributing evenness of species in a biological community, which may eventually lead to the breakdown of an ecosystem [26,27]. Several studies have reported that loss of biodiversity in dental caries is associated with the severity of the disease [28–33]. However, changes in microbial biodiversity remain controversial in periodontitis with some studies reporting loss of biodiversity in disease [34–38] and others indicating the opposite [39–41]. The latter proposed that the increased microbial diversity in periodontitis is due to the increased amount of nutrients derived from host’s tissue degradation in inflammation [39,40]. In addition, there are also reports indicating no significant difference in oral microbial biodiversity between the healthy individuals and the patients with periodontitis [42,43]. The discrepancy of the results may be attributed to differences in studying methods such as sequencing methods, sequencing region, sequencing depth and sampling sites (periodontal pocket depths) [17,39,44].

Another characteristic of dysbiosis is the loss of some beneficial microorganisms [26]. Those species are important for the development and maturation of the local immune system in the oral cavity [19,26]. Those species stimulate the host to generate appropriate immune response, which can protect the host against oral pathogens and from carcinogenic metabolites [45,46]. Moreover, those beneficial microorganisms also participate in the nitrate-nitrite-nitric oxide pathway [47]. Thus, those microorganisms can offer several benefits to the host. Reduction of those beneficial species may weaken the host’s ability to fight against the pathogenic bacteria and predisposing the host to generate an excessive immune response against the host’s own tissue when the host is exposed to carcinogenic metabolites and detrimental vascular changes [19,26]. Loss of beneficial species is an important factor in the development of chronic periodontitis that is persistent and excessive inflammation of periodontal tissues causing the destruction of gingiva, alveolar bone and periodontal ligament and even tooth loss [27].

The most apparent feature of dysbiosis is the overgrowth of pathogenic microorganisms, e.g. the red complex (*Porphyromonas gingivalis, Treponema denticola* and *Tannerella forsythia*) [48]. Although the subgingival microbiome within the healthy periodontal state contains those classically defined oral pathogens, they do not induce any immune responses due to their relatively low abundance in the healthy state [49]. Those microorganisms that do not dominate in the healthy state dramatically increase in periodontitis [17]. But they do not solely cause periodontitis, rather, they synergize to initiate the disease [32]. The recent hypothesis for pathogenesis of periodontitis has been concluded into a Polymicrobial Synergy and Dysbiosis (PSD) model [50,51], which is consistent with the human microbiome studies, the mechanistic studies in animal models and the ‘ecological plaque’ hypothesis, which indicates that environmental factors determine the outgrow of specific pathogenic bacteria, known as pathobionts [32,52,53]. The PSD model proposes that the periodontal disease is not initiated by individual or several causative pathogens but rather by a synergistic polymicrobial community, within which specific constituents, or combinations of functional genes, fulfil distinct roles that converge to shape and stabilize a dysbiotic microbiota, which perturbs host homeostasis [32,54]. For instance, the pathogenic functions require a series of specific molecules instead of a sole molecule. Those molecules include adhesins, receptors, proteolytic enzymes and proinflammatory surface ligands, which cannot be expressed by one species of pathogen. Rather, synergism of the pathogens expressing those molecules sustains a heterotypic and dysbiotic microbial community and acts as a community virulence factor that elicits a non-resolving and tissue-destructive host response [50,55].

## Oral microbiome under DM

Overall, regardless of periodontal health, DM patients exhibited distinct oral microbial features (microbial composition, biological diversity and relative abundance of specific bacteria) compared with healthy controls (Table 1) [56–60]. Based on principal coordinate analysis (PCoA), patients with T2DM and healthy cohorts revealed different oral microbial clusters [56,58]. A clear reduced biological and phylogenetic diversity of oral microbiome was apparent in diabetic and pre-diabetic individuals in comparison with that in the normoglycemic controls [59,60]. The phylum Actinobacteria was present significantly less abundant among patients with T2DM than among the controls [56,57]. Genera *Actinomyces* and *Atopobium* were associated with 66% and 72% decreased risk of diabetes with p-values of 8.9 × 10^–3^ and 7.4 × 10^–3^, respectively [57]. While another study indicated higher abundances of *Actinomyces* and *Selenomonas* with lower abundance of *Alloprevotella* in diabetic patients compared with non-diabetic individuals [58], a subsequent correlational analysis of the differential bacteria and clinical characteristics demonstrated that the oral microbiomes were related to drug treatment for DM and certain pathological changes [60]. But the treatment did not lead to microbial recovery [60]. The subgingival microbiome of diabetics with healthy periodontium reveals lower species richness than non-diabetic healthy controls [60,61], but contains relatively higher abundances of periodontally pathogenic red complex species and a potentially opportunistically pathogenic orange complex and lower abundances of healthy-compatible species [61,62]. This indicates that the subgingival microbiome in diabetic patients with clinically healthy periodontium has a disease-associated community framework, predisposing the individuals to develop periodontal diseases [61–63].

**Table 1.** Recent studies on oral (subgingival) microbiome of diabetic patients (with periodontitis) using NGS

Year	Authors	Study design	Sample size	Groups	Sampling site	Sequencing region	Main findings
2021	Balmasova et al [66]	Cross-sectional	46	Patients with moderate chronic periodontitis (CP) (n = 15) Patients with moderate chronic periodontitis and T2DM (CPT2DM) (n = 15) Healthy subjects (Control) (n = 16)	Four spots of the periodontal pockets/sulcus at the level of the second molars	V3–V4 region and shotgun sequencing for metagenomic analysis	The alpha diversity in the CPT2DM group was increased compared to that in the CP and Control groups. In both groups of patients with periodontitis, the relative abundance of *Porphyromonadaceae* was increased compared to that in the Control group. The CPT2DM group was characterized by a lower relative abundance of *Streptococcaceae*/*Pasteurellaceae* and a higher abundance of *Leptotrichiaceae* compared to those in the CP and Control groups. The CP and CPT2DM groups differed in terms of the relative abundance of *Veillonellaceae* (which was decreased in the CPT2DM group compared to CP) and *Neisseriaceae* (which was increased in the CPT2DM group compared to CP)
2020	Matsha et al [67]	Cross-sectional	128	Normotolerant (n = 32) Prediabetes (n = 32) Screen-detected T2DM (n = 32) Known T2DM receiving treatment (n = 32)	Subgingival crevice between the maxillary second premolar and the first upper molar	V2, V3, V4, V6-V7, V8 and V9 regions of 16S rRNA	The composition of the oral microbiota altered across glycemic statuses as well as different stages of periodontal disease. Of the 9 phyla identified, Firmicutes, Proteobacteria, Bacteroidetes, Fusobacteria and Actinobacteria made up >98% of subgingival microbiome in total subjects. In subjects of diabetes, Fusobacteria and Actinobacteria were significantly more abundant, while Proteobacteria was less abundant. Actinobacteria were markedly reduced, while Bacteroidetes were more abundant in T2DM subjects with gingival bleeding, as compared with T2DM subjects without gingival bleeding. No differences in the abundance of Actinobacteria or Bacteroidetes were observed in diabetics with and without pocket depth (PD) ≥4 mm.
2020	Chen et al [56]	Cross-sectional	442	Adult patients with T2DM (n = 280) Healthy controls (n = 162)	Buccal mucosa	V1–V2 region of 16S rRNA	Patients with T2DM and healthy cohorts exhibited distinct oral microbial clusters based on principal coordinate analysis (PCoA). The Firmicutes/Bacteroidetes ratio increased in T2DM. T2DM patients presented significantly higher numbers of *Neisseria, Streptococcus, Haemophilus* and *Pseudomonas* genera and lower numbers of *Acinetobacteria* compared with healthy controls
2020	Yang et al [60]	Cross-sectional	102	Nondiabetic people (n = 32) Treatment-naïve T2DM patients (n = 31) T2DM patients with metformin treatment (n = 17) T2DM patients with combined medication treatment (insulin plus metformin or other hypoglycemic drugs) (n = 22)	Salivary samples	V3-V4 region of 16S rRNA	The α- diversity was lower in T2DM patients compared with nondiabetic individuals. The β-diversity demonstrated significant differences in the salivary microbiome between the nondiabetic people and patients with a history of diabetes, while little divergence was found among individuals with a history of diabetes. The results revealed changes in the contents of certain bacteria after both the onset and the treatment of diabetes; among these differential bacteria, *Blautia_wexlerae, Lactobacillus_fermentum, Nocardia_coeliaca* and *Selenomonas_artemidis* varied in all processes. Salivary microbiomes were related to drug treatment and certain pathological changes. T2DM could significantly alter the salivary microbiota, while treatment did not lead to flora recovery.
2019	Farina et al [65]	Cross-sectional	12	Patients with periodontitis and T2DM (T2D+ P+ group) (n = 3) Patients with periodontitis but not T2DM (T2D− P+ group) (n = 3) Patients with type 2 diabetes but not periodontitis (T2D+ P− group) (n = 3) Patients without either T2DM or periodontitis (T2D− P− group) (n = 3)	Subgingival plaques without bleeding on probing in P- patients; subgingival plaques at the deepest probing depth among those positive to bleeding on probing in P+ patients.	Metagenomic shotgun sequencing	The presence of T2DM and/or periodontitis was associated with a tendency of the subgingival microbiome to decrease in richness and diversity. The presence of type 2 Diabetes Mellitus was not associated with significant differences in the relative abundance of one or more species in patients either with or without periodontitis. The presence of periodontitis was associated with a significantly higher relative abundance of *Anaerolineaceae bacterium oral taxon* 439 in type 2 Diabetes Mellitus patients.
2019	Shi et al [62]	Longitudinal	31	T2DM patients with Periodontitis (n = 8) T2DM patients with healthy periodontium (n = 7) Nondiabetic subjects (ND) with periodontitis (n = 8) Nondiabetic subjects (ND) with healthy periodontium (n = 8)	Subgingival plaques	Metagenomic shotgun sequencing	There was no significant difference in the subgingival microbiome between the resolved state (after periodontal treatment) and the healthy state in either T2DM or ND. In T2DM patients, the microbiome in the periodontitis state was not significantly different from that in the resolved state or the healthy state. The shift of subgingival microbiome from the healthy state to the periodontitis state was less prominent in T2DM compared with ND subjects, yet the clinical signs of disease were similar for both. Highly correlated presence of pathogenic species in relative abundance was not only in the periodontitis state but also in the healthy state in T2DM. A set of microbial marker genes associated with the clinical states were identified. These identified genes were significantly enriched in 21 pathways, some of which are associated with periodontitis and some potentially link T2DM and periodontitis.
2019	Saeb et al [59]	Cross-sectional	44	T2DM patients (n = 15) Impaired glucose tolerance (IGT) subjects (n = 10) Control subjects (n = 19)	Saliva samples	V2, V3, V4, V6, V7, V8 and V9 regions of 16S rRNA	A clear reduction of the number of species was observed in both IGT and T2DM groups compared with that in the control group. The phylogenetic diversity (PD-SBL) value in the normoglycemic group was higher than that in the diabetes group. The T2DM group exhibited the highest evenness value and the highest bacterial pathogenic content.
2018	Longo et al [68]	Cross-sectional	21	T2DM non-smoking patients with chronic periodontitis who had inadequate glycemic status (DMI-HbA1c≥8%) (n = 11) T2DM non-smoking patients with chronic periodontitis who had adequate glycemic status (DMA-HbA1c<7.8%) (n = 11)	Mesio-buccal sites of teeth with medium (PD 4–6 mm) and deep pockets (PD ≥7 mm) in two contralateral quadrants	V5-V6 region of 16S rRNA	Microbiome in the DMA group presented higher diversity than that in the DMI group. Inadequate glycemic control favored fermenting species, especially those associated with propionate/succinate production, whereas those forming butyrate/pyruvate were decreased.
2017	Long et al [57]	Cross-sectional		Participants with incident diabetes (n = 98) Obese non-diabetics (n = 99) Normal weight non-diabetics (n = 97)	Mouth rinse samples	V4 region of 16S rRNA	The phylum Actinobacteria and almost all taxa in this phylum were present significantly less abundant among patients with diabetes (median abundance of 5.1%) than among the controls (median abundance of 6.8%) (*p* = 3.9 × 10^−3^). This phylum Actinobacteria was also less abundant among non-diabetic obese subjects compared to normal weight individuals, particularly genera *Mobiluncus, Corynebacterium* and *Bifidobacterium.*
2017	Ganesan et al [61]	Cross-sectional	175	Normoglycemic non-smokers with periodontitis (n = 25) Hyperglycemic non-smokers with periodontitis (n = 25) Normoglycemic smokers with periodontitis (n = 25) Hyperglycemic smokers with periodontitis (n = 25) Normoglycemic non-smokers with healthy periodontium (n = 25) Hyperglycemic non-smokers with healthy periodontium (n = 25) Normoglycemic smokers with healthy periodontium (n = 25)	Deep (diseased) sites and shallow (healthy) sites of subjects with periodontitis; randomly selected interproximal sites in periodontally healthy subjects	V1–V3 and V7–V9 regions of 16S rRNA	In subjects with periodontitis, differences in the microbial community structure or membership were not apparent between deep (diseased) sites and shallow (healthy) sites. Diabetic patients with periodontitis showed different microbial assemblages compared with normoglycemic patients with periodontitis, and significant clustering was observed based on HbA1c levels (pre-diabetic (<6.5%), diabetic (6.5–9.9%) and diabetic (>10%) within the diabetic group). Diabetics with periodontitis showed a significant lower species richness, lower levels of anaerobes (*Treponema, Porphyromonas, Prevotella* and *Parvimonas*) and higher levels of facultatives (*Lactobacillus, Corynebacterium* and *Pseudomonas*) compared to normoglycemic individuals with periodontitis. Periodontally healthy diabetics exhibited significantly lower species richness than both diabetics with periodontitis and periodontally healthy controls. Periodontally healthy diabetics presents a disease-associated community framework (decreases in relative abundances of health-compatible species and increases in levels of species belonging to the genera *Porphyromonas, Prevotella, Campylobacter* and *Fusobacterium*).
2017	Ogawa et al [58]	Cross-sectional	24	T2DM elderly residents (n = 3) Non-T2DM elderly residents (n = 12) Young healthy controls (HC) (n = 9)	Salivary samples	Sequencing V4 region of the 16S rRNA gene	The a-diversity, in terms of operational taxonomic unit richness, was higher in the non-T2DM group than in the HC group. Weighted UniFrac distance analysis showed that the salivary microbial communities in T2DM were separately clustered from those in the non-T2DM and HC groups. In the T2DM group, *Actinomyces* and *Selenomonas* showed significantly higher abundance, whereas *Alloprevotella* showed significantly lower abundance, relative to the non-DM group.
2013	Zhou et al [63]	Cross-sectional	31	Non-diabetic subjects without periodontitis (n = 5) Non-diabetic subjects with periodontitis (n = 6) T2DM patients without periodontitis (n = 8) T2DM patients with periodontitis (n = 12)	Subgingival plaque samples from the deepest sites of the molars	V1-V3 region of 16S rRNA	Comparing periodontally healthy samples with periodontitis samples identified 20 health-associated and 15 periodontitis- associated OTUs. In the subjects with healthy periodontium, the abundances of three genera (*Prevotella, Pseudomonas* and *Tannerella*) and nine OTUs were significantly different between diabetic patients and their non-diabetic counterparts. In the subjects carrying periodontitis, the abundances of three phyla (Actinobacteria, Proteobacteria and Bacteriodetes), two genera (*Actinomyces* and *Aggregatibacter*) and six OTUs were also significantly different between diabetics and non-diabetics.
2012	Casarin et al [64]	Cross-sectional	23	Subjects with uncontrolled T2DM (glycated hemoglobin > 8%) presenting severe and generalized chronic periodontitis (n = 12) Nondiabetic subjects presenting severe and generalized chronic periodontitis (n = 11)	The bottom of the periodontal pockets	Partial sequence (600bp) of 16S rRNA	Subjects with uncontrolled T2DM and chronic periodontitis harbored a significantly different microbiota in periodontal pockets compared with non-diabetic subjects. Diabetic subjects presented higher percentages of total clones of *Saccharibacteria, Aggregatibacter, Neisseria, Gemella, Eikenella, Selenomonas, Actinomyces, Capnocytophaga, Fusobacterium, Veillonella* and *Streptococcus* genera and lower percentages of *Porphyromonas, Filifactor, Eubacterium, Synergistetes, Tannerella* and *Treponema* genera than nondiabetic individuals (p < 0.05). Some phylotypes, such as *Fusobacterium nucleatum, Veillonella parvula, V. dispar* and *Eikenella corrodens* were detected significantly more often in diabetic subjects than in nondiabetic subjects.

In diabetic patients with periodontitis, oral microbial profiles also reveal distinct features compared with non-diabetic patients with periodontitis (Table 1) [60,64,65], with reported either increased [66,67] or reduced microbial diversity [61]. The subgingival microbiome of diabetic patients with periodontitis exhibited relatively higher abundances of *Leptotrichiaceae, Neisseriaceae* and *Dialister; Lactobacillus, Corynebacterium* and *Pseudomonas; Saccharibacteria, Aggregatibacter, Neisseria, Gemella, Eikenella, Selenomonas, Actinomyces, Capnocytophaga, Fusobacterium, Veillonella, Streptococcus* and *Actinomyces* and relatively lower abundances of *Filifactor, Treponema, Porphyromonas, Prevotella*, and *Parvimonas* than non-diabetic patients with periodontitis, while both groups showed similar clinical manifestations [61,63,65,66]. Furthermore, the difference of subgingival microbiome between the periodontitis state and the healthy state in diabetic patients was less prominent than that in non-diabetic patients, since the difference of subgingival microbiome of the disease state and the healthy state in T2DM patients could not be clearly distinguished [62]. It should be mentioned that the pocket depth and bleeding had no significant impact on the subgingival microbiome in diabetic patients [61,66,68], which is different from the cases of non-diabetic patients with periodontitis [39,62]. After treatment (in the resolved state), the subgingival microbiome of both T2DM patients and non-diabetic individuals resembles the subgingival microbiome in the healthy state [62]. However, in the resolved state, the subgingival microbiome of T2DM patients revealed lower abundances of orange complex and red complex species than non-diabetic individuals, which indicates that T2DM patients after periodontal treatment are less tolerant to the periodontitis-associated species and thus have lower periodontal pathogens to maintain the clinically resolved periodontal health [62].

To confirm the effect of DM on oral microbiome and its association with periodontitis, an animal study was carried out by Xiao et al [69]. In this study, the oral microbiome of two groups of mice (one was prone to develop DM and the other was normal littermates) presents similar oral microbiome at the beginning, but revealed apparently different after one group became diabetic [69]. DM reduced the diversity of total oral microbiome in mice but increased the levels of Proteobacteria (*Enterobacteriaceae*) and Firmicutes (*Enterococcus, Staphylococcus* and *Aerococcus*) [69], which was associated with pathologic changes reported in other studies [14]. Furthermore, by transferring the oral microbiota of diabetic and non-diabetic mice to germ-free recipient mice, greater periodontal inflammation and bone loss were observed in the mice receiving bacteria from diabetic mice [69]. Moreover, the same group also found that IL-17 played an important role in the oral microbial alteration of diabetic mice, as local inhibition of IL-17 could not only render the oral microbiome of diabetic mice similar to that of non-diabetic mice but also reduce the pathogenicity of oral microbiota in diabetic mice [69]. However, since the oral microbiome of mice does not share similarity to human oral microbiome, whether these findings are suitable for human requires further studies [70,71].

Further studies indicated that prediabetic and diabetic periodontitis patients with different glycemic levels may harbor different subgingival microbiome [61,67,68]. Subgingival microbiome in periodontitis patients with inadequate glycemic levels (HbA1c ≥ 8%) revealed a reduced biodiversity compared to patients with adequate glycemic levels (HbA1c < 7.8%) [68]. Levels of recognized periodontopathogens, such as *Porphyromonas gingivalis, Tannerella forsythia* and *Treponema denticola* that are indicative of periodontitis, did not differ between diabetic patients with different glycemic levels [68]. Higher abundances of species that are able to use carbohydrates or their by-products within families *Streptococcaceae, Prevotellaceae* and *Veillonellaceae* were seen in patients with a higher glycemic level, whereas those forming butyrate/pyruvate were decreased in patients with inadequate glycemic control [68]. The increased level of *S. agalactiae* in diabetic patients with an inadequate glycemic level belongs to Group B streptococci (GBS) and is an important invasive pathogen in newborn infants, elderly and those with chronic diseases [68]. *Prevotella* produces acetate and succinate from glucose fermentation, and the cultivable species *Alloprevotella* are saccharolytic, producing acetate and major amounts of succinate [68,72,73]. Their growth in liquid media is stimulated by fermentable carbohydrates [72]. The increase of *Veillonellaceae* in diabetic patients with periodontitis was due to the utilization of fermentation catabolites produced by the fermenting species [68]. This group of bacteria convert lactate and succinate to acetate and propionate and may play an active role in reducing the environment acidity [74,75].

As periodontitis is driven by polymicrobial effects, microorganisms may harbor redundant roles contributing to the development of periodontitis. Thus, detecting the changes of microbial pathways in DM may provide more intrinsic information about how DM predisposes patients to develop periodontitis. In both diabetic and nondiabetic patients with periodontitis, 4 functional pathways of virulence factors were enriched [62]. They are pathways associated with cell motility (bacterial motility, flagellar assembly and bacterial chemotaxis) and a signal transduction pathway (two-component system) [62]. In addition, three pathogenic pathways were less prevalent in T2DM patients with periodontitis than nondiabetic patients with periodontitis [62]. There are two pathways in lipid metabolism (ether lipid metabolism and arachidonic acid metabolism) and one pathway in carbohydrate metabolism (inositol phosphate metabolism), which are linked via lipoprotein-associated phospholipases, a group of inflammatory enzymes associated with oral infections [62]. In contrast, three pathways of carbohydrate metabolism (butanoate metabolism, pentose and glucuronate interconversions, and ascorbate and aldarate metabolism) were more prevalent in T2DM than in non-T2DM in both the periodontitis state and the healthy state [62]. The ascorbate and aldarate metabolism pathway has been associated with inflammatory diseases including periodontitis [76,77] and T2DM [78]. Microbial butanoate metabolism has been indicated as a metabolic signature of periodontal inflammation [79], and butyrate can influence insulin sensitivity [80]. These results might account for the epidemiological studies, which support that periodontal infection has an adverse effect on glycemic control [62].

Taken together, recent studies on oral microbiome in diabetics using NGS have provided great information about the oral microbial alteration under DM (Table 1), which would contribute to understanding the inter-relationship between periodontitis and DM.

## Changes of periodontal immune status in DM

Diabetic complications are frequently linked to increased inflammation, and substantial evidence has indicated that DM increases the inflammation of periodontal tissues. Both T1DM and T2DM lead to increased expression of inflammatory cytokine and chemokine in human periodontal tissues [81,82]. Those cytokines include tumor necrosis factor, prostaglandin E2, interleukin-1beta, interleukin-17, interleukin-23 and interleukin-6 [83,84]. On the contrary, anti-inflammatory factors such as interleukin-4, interleukin-10, transforming growth factor-beta and anti-inflammatory lipid-based mediators are reduced in diabetics, along with reduction of anti-inflammatory regulatory T cells and M2 macrophages [85–87].

The increased expression of inflammatory cytokines and chemokines in diabetics leads to increased vascular permeability and recruitment of inflammatory cells in response to bacterial challenge or other pro-inflammatory stimuli [83,88,89]. Moreover, the behaviors of leukocytes are altered under DM [90]. For instance, DM reinforced the interaction between neutrophils and gingival endothelial cells by increasing the expression of P-selectin glycoprotein ligand-1 and CD11a on neutrophils and P-selectin expression on endothelial cells [89]. Studies of other diabetic complications indicate that DM increases pro-inflammatory M1 macrophage polarization [91,92]. The functions of monocytes and macrophages in patients with DM are influenced by their interactions with the local environment within periodontal tissues, including interactions between receptor for advanced glycation end products (RAGEs) and advanced glycation end products (AGEs), toll-like receptor signaling, reactive oxygen species (ROS) production and others [90].

High glucose content can lead to the formation of advanced glycation end products (AGEs), which are products of irreversible non-enzymatic glycation and glycoxidation of proteins, including lipoproteins, intracellular proteins and plasma proteins [90,93–95]. The excessive accumulation of AGEs can alter cytoplasmic and nuclear factors and induce the formation of stable abnormal cross-links on collagen that changes its structure and function [96]. AGEs also increase the expression of receptor for advanced glycation end products (RAGEs) in gingiva [90,97]. The interaction between AGEs and RAGEs can modulate cell behavior and inflammation in periodontal tissues. For instance, the binding of AGEs and RAGEs on gingival fibroblasts can activate nuclear factor-kappa B, which induces the expression of inflammatory cytokines such as interleukin-6 and tumor necrosis factor and stimulates the production of ROS [98]. Although RAGEs signaling does not directly initiate inflammation, it perpetuate and amplify the responses of monocytes, macrophages, neutrophils, endothelial cells and chondrocytes in the context of inflammatory processes, diabetes complications and atherosclerosis [99]. Interference with RAGEs signaling under chronic inflammatory conditions results in improvement in clinical and biochemical signs of inflammation, including suppression in periodontitis-associated bone loss and decreased generation of the proinflammatory cytokines tumor necrosis factor-α and interleukin-6 in gingival tissues [100].

Reactive oxygen species (ROS) including free radicals (e.g. superoxide O ˙_2_^−^ and hydroxyl radicals ˙OH), nonradical oxygen species [e.g. hydrogen peroxide (H_2_O_2_)] and reactive lipids are generated by cellular functions such as phagocytosis and mitochondrial cell respiration. An overproduction of superoxide by the mitochondrial electron transport chain is considered as the unifying underlying pathological mechanism of diabetic complications [93]. Chronic exposure to high glucose content induces the production of higher levels of ROS that may cause damage to DNA and structural components of cells and cell apoptosis [101]. ROS may also activate mitogen-activated protein kinase and nuclear factor-kappa B signaling, resulting in the production and release of multiple inflammatory factors [83,102]. Diabetic patients have an increased number of inducible nitric oxide synthase-positive cells in the periodontium, and levels of lipid peroxides are elevated in the gingival crevicular fluid of T2DM patients [81,103]. There is a significant correlation between lipid peroxidation and periodontal inflammation in T2DM patients, which suggests that lipid peroxides may contribute to more severe periodontal inflammation [81,83,103]. Moreover, antioxidant capacity is reduced in diabetic patients, which also contributes to the increased levels of ROS and their adverse impact on periodontium indirectly [83].

Toll-like receptors represent an important mechanism by which the host detects a variety of invading microorganisms and trigger the expression of genes that control the innate immune system when binding to their ligands such as LPS, bacterial DNA, double-stranded RNA, peptidoglycans and lipoproteins [90]. There is some evidence for possible alterations in toll-like receptor expression in the gingival tissues of patients with DM [90]. Elevated expression of toll-like receptor 2 and toll-like receptor 4 is found in gingival tissue biopsies from patients with DM and periodontitis compared with patients with periodontitis alone [83,104]. In vitro studies also indicated increased expression of toll-like receptor 2 in human gingival fibroblasts cultured with high levels of glucose [105].

An important part of the periodontal immune system that has not received much attention is the gingival epithelial barrier. Gingival epithelium serves as an effective barrier in protecting the gingival connective tissue from oral or subgingival microorganisms [106]. The structure and function of gingival epithelium may also be altered by DM since the keratinocytes are affected by DM [107,108]. But the actual impact of DM on barrier function requires further studies to be fully understood.

## Mechanisms that DM affects oral microbiome

While recent evidence indicates that DM can alter the oral microbiome, specific mechanisms are not fully understood. According to the ecological plaque hypothesis, changes of the microbiome may be determined by the inherent characteristics of the microbial environment such as oxygen tension, pH, redox potential and nutrition supply [109]. Thus, the oral environment may act as filters to select species that holds suitable habitat within this environment. Besides, the formation of microbial communities also results from interactions of the community members with different metabolic, structural and nutritional traits.

In this context, the inflammation imposes a strong ecological selective pressure that drives dysbiosis with the expansion of periodontitis-associated microbial species at the expense of health-compatible species [32,110]. Destruction of periodontal tissues induced by the inflammation generates substances such as collagen fragments and heme-containing compounds, which could be sources of amino acids and iron for those microbial species that utilize them [111]. Those substances can be transferred via increased gingival crevicular fluid into the gingival crevice, fostering the outgrowth of proteolytic and asaccharolytic microbial species with iron-acquisition capacity, such as *P, gingivalis*, which can uncouple the nutritionally favorable inflammatory response from microbicidal responses [112]. Moreover, inflammation causes low redox potential that favors the development of anaerobic bacteria [110]. Consistently, a community-wide transcriptomic study on periodontitis-associated subgingival microbiome has indicated elevated expression of proteolysis-related genes, genes for peptide transport and acquisition of iron, as well as the genes associated with synthesis of lipopolysaccharides that would enhance the proinflammatory ability of the microbiota [113]. Those species that capitalize on the inflammatory spoils and expand their populations are termed ‘inflammophilic pathobionts’ [111]. Studies also indicated the selective overgrowth of pathobionts by addition of serum, hemoglobin or hemin to the in vitro formed oral multi-bacterial community [114]. Those pathobionts upregulate the expression of virulence-associated genes that encode proteases, hemolysins and proteins associated with hemin transport [114].

**Figure 1.**
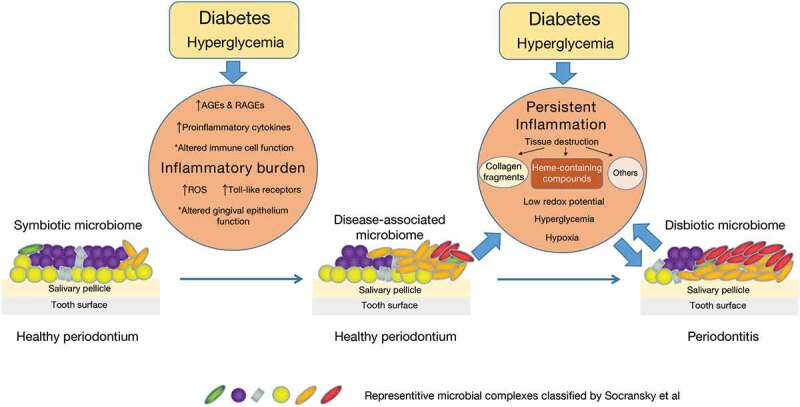
The plausible mechanism of DM altering oral microbiome.

Together, all these findings support hypothesis that the selective pressure induced by the inflammatory environment drives the dysbiosis of the oral microbiome. Certain microorganisms (inflammophilic pathobionts) that thrive under inflammatory conditions stimulate greater inflammation, leading to reciprocal reinforcement between dysbiosis and inflammation. This vicious cyclic process persists and finally causes the clinical manifestations of periodontitis, e.g. attachment loss and periodontal pocket. As mentioned above, DM imposes a pre-existing inflammatory burden on periodontal tissues with increased levels of cytokines and ROS, altered immune cell function, accumulation of AGEs and upregulated expression of RAGEs and toll-like receptors. This pre-existing inflammatory burden and the increased level of glucose in gingival crevice fluid under DM foster the growth of inflammophilic pathobionts including the red complex, orange complex and the pathobionts that metabolize carbohydrates or their by-products, thus causing the oral microbiome to become a disease-associated community framework although the periodontium seems healthy (Figure 1). This dysbiotic microbiome initiates greater inflammation in turn, finally leading to the development of periodontitis (Figure 1).

## Discussion

Since the first observation of microbes in dental plaque by Antony van Leeuwenhoek using his own microscope almost four hundred years ago, the role of oral microbial species has evolved from being simply considered as ‘passengers’ on the human host to one important factor that can modulate the health of their human host and induce diseases by working as a whole through inter-microbial synergism, signaling or antagonism, instead of by one or several species. The pathogenesis of periodontal disease has been explained by the ‘Polymicrobial Synergy and Dysbiosis (PSD)’ hypothesis, which suggests that the oral microbiome shifts from a healthy state to a diseased state in periodontal disease. Although the reasons for this shift are still not fully understood, ecological studies have shed light on this, indicating that environmental changes, such as pH, oxygen level and nutrition supply in specific oral sites, might drive this microbial alteration.

A mass of data has indicated that patients with DM or poorly glycemic control are more risky to periodontitis, which urges the studies on the oral microbiome in diabetics. The results revealed the alterations in the oral microbiome of subjects with DM or undesired glycemic control, although there were tiny differences in the specific microbial changes within different studies. Also, these findings support the notion that a dysbiotic oral microbiome is a plausible contributory factor in the pathogenesis of diabetes-induced periodontal disease. Since diabetic complications are frequently linked to increased inflammation, changes in the immune status of periodontal tissues in DM play an important role in the oral microbial shift in diabetics. Based on the studies of interactions between host and microbial community or certain microorganisms, this article discusses the mechanism of microbial change in DM that the selective pressure induced by inflammatory environment drives the dysbiosis of the oral microbiome, which in turn enhanced the inflammation. As the vicious cyclic process persists, periodontitis occurs.

There are also shortcomings about this review. First, this article is a narrative review. Although narrative review can be evidence-based, biased point of view may be possibly included in a narrative review. Nevertheless, the authors of this article hope that this review could serve as a valuable resource for interested readers to further explore the literature in periodontal microbial etiology and DM. Second, this article does not discuss the relationship between oral microbial shifts and the microbiome in other sites of digestive tract under DM. Since the oral cavity is a part of digestive tract, changes of intestinal microbiome in DM might have an influence on oral microbiome or vice versa. However, the relationships between intestinal microbiome and DM is complex, which may be beyond the focus of this review.

## Future direction

While the collective analysis of those studies in this field has provided valuable insights, mechanistic and large longitudinal studies as well as intervention trials are still in need to confirm these results. This information will contribute to identification of microbial markers of disease activity, recurrence and responses to different types of treatment [115]. Furthermore, knowing the deleterious effects of the modulation of the periodontal microbiome by diabetes, it becomes important to investigate whether it is possible to reverse these dysbiotic ‘at-risk’ microbial shifts, returning it to hemostasis with therapeutic approaches [115]. There are several new directions that have potential to fill current gaps in this field. In addition to microbiome, other omics such as metabolomics, virome, mycobiome, proteomics and host genomics provide new methodologies to analyze the oral health/disease puzzles by incorporating them into bioinformatics models. As studies have demonstrated that microbial alterations in our body correlate with numerous diseases, manipulation of the microbial communities (via transplants, probiotics or targeted drug delivery) could be used to treat disease. In vitro and in vivo/preclinical studies have shown promise for applying probiotics in treating periodontal disease [116]. Moreover, with the development of large data sets that comprise microbial and clinical information, and the application of robust bioinformatics approaches, it is potentially to make really personalized dentistry in periodontology according to the metadata of individual subjects.
